# Young Adults as a Tobacco Control Priority Population in the US

**DOI:** 10.1001/jamanetworkopen.2020.19365

**Published:** 2020-10-01

**Authors:** Ollie Ganz, Cristine D. Delnevo

**Affiliations:** Rutgers Center for Tobacco Studies, Rutgers Biomedical and Health Sciences, New Brunswick, New Jersey; Department of Health Behavior, Society and Policy, Rutgers School of Public Health, Piscataway, New Jersey.

The study by Barrington-Trimis et al^1^ used data from the National Survey on Drug Use and Health to examine the proportion of young adult cigarette smokers (aged 22-23 years) who initiated smoking in young adulthood from 2002 to 2018. Barrington-Trimis and colleagues^1^ found that during this period with population-level declines in cigarette smoking among youth and young adults, the average age of smoking initiation and the proportion of new initiates and daily smokers who were young adults increased. This shift is not surprising given that the prevalence of cigarette smoking decreased substantially among adolescents aged 12 to 17 years from 13.0% in 2002 to an all-time low of 2.7% in 2018.^2^

Historically, the overwhelming majority of cigarette smokers started during adolescence. Indeed, the 2012 Surgeon General’s report on youth tobacco use found that nearly 9 of 10 smokers started smoking by age 18 years.^3^ However, the study by Barrington-Trimis et al^1^ builds on on an emerging body of evidence suggesting that initiation of cigarette smoking is shifting from adolescence to young adulthood.^3^ Research from the early 2000s examined delayed initiation as a potential explanation for increases in young adult cigarette smoking prevalence observed in the 1990s.^4^ More recently, a study by Cantrell et al^5^ found that from 2002 to 2015, cigarette initiation rates among young adults (aged 18-21 years) surpassed that of youth (aged 15-17 years) and remained higher in 2015, despite declines in initiation for both groups beginning in 2009. Cantrell et al^5^ also found that the incidence of daily smoking was higher among young adults compared with youth across the study period.

As Barrington-Trimis et al^1^ point out, this shift in initiation from adolescence to young adulthood over the last 2 decades occurred within the context of massive tobacco control efforts to reduce cigarette smoking initiation among youth specifically, beginning with the 1998 Master Settlement Agreement (MSA), an agreement between the major tobacco manufacturers and 46 states in the US to recover the health-related costs to the states caused by cigarette smoking. The MSA imposed multiple restrictions on tobacco industry marketing, most notably the prohibition of direct and indirect marketing to youth. The MSA also created a national tobacco control organization, the American Legacy Foundation (now known as Truth Initiative), which developed the truth campaign, a national youth smoking-prevention campaign that launched in 2000 and still exists today. Since the launch of the truth campaign, youth (aged 12-17 years) smoking prevalence has descreased and evaluation findings suggest that the campaign prevented hundreds of thousands of youth from initiating cigarette smoking.^6^ Additionally, in 2009, President Obama signed the Family Smoking Prevention and Tobacco Control Act, which granted the US Food and Drug Administration (FDA) the authority to regulate tobacco products and established the FDA Center for Tobacco Products. As part of their mission to improve public health, the Center for Tobacco Products has launched several youth tobacco prevention public education campaigns. Most notably, the FDA’s The Real Cost campaign may have prevented an estimated 348 398 young people (aged 11-18 years) in the US from initiating cigarette smoking from February 2014 to March 2016.^7^

As a result of the MSA, young adults became the youngest age group that the tobacco industry could legally target. Even prior to the MSA, however, young adults were an important customer base for the tobacco industry; a tobacco company infamously referred to young adults as “replacement smokers” for those who quit smoking or died.^3^ After the MSA, tobacco industry marketing and promotional efforts targeting young adults only intensified.^3^

The shift toward initiation of cigarette smoking in young adulthood has resulted in the emergence of young adult–focused tobacco control interventions in recent years. In 2014, the truth campaign began focusing on young people aged 15 to 21 years. Similarly, the FDA’s This Free Life public education campaign, which launched in 2016, was designed to prevent and reduce tobacco use among lesbian, gay, bisexual, and transgender young adults (aged 18-24 years). Additionally, a recently updated systematic review^8^ of smoking cessation interventions for young adults identified 3 promising smoking-cessation strategies for this population that emerged since the prior review in 2010. More recently, following rapid diffusion of numerous states and localities raising the minimum age for sale of tobacco products from 18 to 21, Congress followed suit in December 2019 and raised the minimum legal age of sale nationally to 21 years.

The findings from Barrington-Trimis et al^1^ highlight an emerging need for tobacco control efforts to further focus on reducing cigarette smoking among young adults. In particular, the increase in the proportion of daily smokers who are young adults is concerning, as it suggests that young adults are not just experimenting with cigarettes. Rather, many are progressing to established patterns of use, defined by lifelong addiction and elevated risk for tobacco-related morbidity and mortality.^3^ This escalation from experimentation to daily smoking highlights the need for what some have termed *prevescalation* interventions for young adults that specifically focus on interrupting transitions to more established and dependent patterns of smoking.^9^

Finally, although Barrington-Trimis and colleagues^1^ raise valid concerns about the increasing proportion of new initiates and daily smokers who are young adults, we think it is important to recognize that these findings are the result of a larger public health success of dramatic reductions in youth and young adult smoking. It is also important to recognize that tobacco use patterns are increasingly diverse, and tobacco control interventions must consider how to maintain these historically low levels in cigarette smoking and reduce them even further. We agree with the authors that expanding tobacco control efforts to be more inclusive of young adults is needed and also encourage the development of novel strategies to reduce cigarette use among this population, such as interventions designed to prevent progression to more established patterns of use.^10^
